# School Demands and Coping Resources−Associations with Multiple Measures of Stress in Mid-Adolescent Girls and Boys

**DOI:** 10.3390/ijerph15102143

**Published:** 2018-09-29

**Authors:** Viveca Östberg, Stephanie Plenty, Sara B. Låftman, Bitte Modin, Petra Lindfors

**Affiliations:** 1 Department of Public Health Sciences, Stockholm University, SE-106 91 Stockholm, Sweden; sara.brolin.laftman@su.se (S.B.L.); bitte.modin@su.se (B.M.); 2 Institute for Futures Studies, Box 591, SE-101 31 Stockholm, Sweden; stephanie.plenty@iffs.se; 3 Swedish Institute for Social Research, Stockholm University, SE-106 91 Stockholm, Sweden; 4 Department of Psychology, Stockholm University, SE-106 91 Stockholm, Sweden; pls@psychology.su.se

**Keywords:** school, demands, control, support, coping, stress, biomarkers

## Abstract

Stress, and stress-related health complaints, are common among young people, especially girls. Since studies have shown that school demands are an important driver of stress in adolescents, identifying if school-based resources can protect against stress is highly relevant. The aim of this study was to analyse task-related demands and task-related coping resources as aspects of the school work environment of potential relevance for stress in mid-adolescent girls and boys. The data came from “The School Stress and Support study” (TriSSS) conducted among students in grades 8 and 9 (aged 14–16 years). Self-reports of demands, coping resources, stress, as well as recurrent pain, were collected through questionnaires (*n* = 411). A subsample of students (*n* = 191–198) also provided salivary samples, which were analysed for the stress marker cortisol. Linear (OLS) and binary logistic regression analyses showed that higher demands were associated with more perceived stress, a higher likelihood of recurrent pain, and a lower cortisol awakening response. Greater coping resources were associated with less perceived stress and a lower likelihood of recurrent pain, but there was no association with cortisol. The strength of the associations differed by gender. The findings suggest that schools can promote student wellbeing by providing clear and timely information and teacher support to the students, especially for boys. Identifying specific features of the schoolwork that give rise to stress and to modify these accordingly is also of importance, especially for girls.

## 1. Introduction

Stress is commonly defined as the imbalance between stressors, i.e., experienced demands or challenges, and an individual’s capacity to deal with these [1]. Whether or not demands or challenges do in fact act as stressors therefore depends on the individual’s interpretation [1,2], as well as on available coping resources [3]. Short-term or acute stress is typically an adaptive response whereas long-term or chronic stress is known to have detrimental effects on mental and physical health [4].

A large share of adolescents in Western countries, especially girls, report high levels of stress-related health complaints [5,6]. The causes behind stress are complex and many factors play a role. One area of central importance is the school and the psychosocial working conditions it provides for the students. Numerous studies have identified school demands as an important driver of stress among adolescents [7,8,9,10,11,12,13,14,15], with health implications particularly among girls [16]. Thus, investigating the potential of school-based resources to counteract stress at school is highly relevant.

Stress can be conceptualised and measured in several ways. For instance, stress can be measured as the individual’s subjective experience of an imbalance, i.e., perceived stress. Stress can also be reflected in recurrent pain suggesting physiological consequences of the stress process. In both these cases information can be gathered through self-reports in questionnaires. However, stress can also be investigated via biomarkers. For example, the hormone cortisol reflects the activation of the hypothalamic-pituitary-adrenocortical (HPA) axis, which is involved in the physiological stress response, with salivary cortisol being a commonly used biomarker of stress-related functioning [17,18,19]. Studies of cortisol rhythms in general populations of adolescents are few [17,18,20]. Similar to adults, existing research on adolescents show that salivary cortisol reflects long-term adjustment and chronic stress-related HPA-axis functioning. Responses to acute stress can be adaptive, while chronic stress is considered detrimental and can lead to HPA dysregulation [21], which may manifest itself in increased or decreased cortisol levels [22,23]. To obtain a broader view of stress among adolescents, it is recommended that researchers use multiple methods of measuring stress, including the collection of both survey/interview data and of stress hormone measures [24]. 

In order to map stressful conditions at work in the adult population the ‘demand-control model’, and the extended ‘demand-control-support model’, has frequently been used [25]. The model proposes that working conditions involving high demands may induce stress while working conditions offering individuals control over how to deal with these demands may reduce the likelihood of stress. Moreover, control and social support are conceptualised as two types of coping resources that can help individuals manage their work demands. Thus, the likelihood of high demands resulting in stress may be counteracted by a higher degree of control, and/or social support. The model has also been applied to adolescents and their working conditions in school. In empirical studies of health among students, however, the effect of control on stress in terms of decision authority has often been absent or unclear [11,12,26,27], and few studies have found any buffering effect [7,8]. Thus, control in terms of decision authority seems to be of limited importance for protecting against stress associated with school. 

While the measurement of demands commonly captures the student’s own situation and experience, the measurement of control often has focussed on students’ participation in overall decision making rather than on individual resources to deal with specific situations or tasks. Moreover, the control dimension may cover other areas than the stressor (i.e., task-related demands), such as whether a class is allowed to have a say in decisions regarding class rules, or which topics to study/work on. Thus, if personal control is central, any effects of such control will be difficult to identify. Additionally, if the matching between type of stressors and type of coping resources is important for preventing stress it will be difficult to find any relevant combinations of demands and control. A similar reasoning applies to social support within the school setting: Even though social support per se has been associated with student health, its measurement has often been broad and included various types of support (e.g., emotional, instrumental, and informational support) and/or multiple sources of support simultaneously (e.g., teachers, parents, and classmates). Again, if the matching between a stressor and support matters, buffering effects of support will be difficult to establish [12,28]. 

The present study focused on task-related school demands and key resources for coping with these demands in order to examine whether such resources can protect against stress in students. With regard to coping resources, we consider students’ personal control and support from teachers as resources provided by the school, through the teachers. All studied aspects will thus be traceable to the organisation of the school and reflect aspects of the psychosocial work environment provided for students. Since stress-related health complaints are more common among girls than among boys [5,6], and since school demands are especially important for girls’ health [16], we also investigate gender differences in any associations between demands, coping resources and stress.

The aim of the present study was to analyse task-related demands and task-related coping resources as aspects of the school work environment that are of potential relevance for stress experiences among mid-adolescent girls and boys. We used a multiple methods data collection where stress was measured through self-reports of perceived stress and recurrent pain, and also through salivary cortisol. The research questions were as follows:

1. Is the level of demands, the level of coping resources, and the combination of demands and coping resources associated with different measures of stress in students?
2. Are there any differences between girls and boys?

## 2. Materials and Methods 

### 2.1. Participants and Procedure

Data came from “The School Stress and Support study” (TriSSS) conducted in 2010. Students in the 8th and 9th grades (aged 14–16 years, *n* = 545) from two compulsory schools in Stockholm were invited to participate in a study of students’ daily life, stress, and health. This was a multiple methods study with data collected through questionnaires, saliva sampling and personal interviews. In the Swedish school system, the 8th and 9th grades are the two final years of the compulsory school. One school was located in the city centre and the other one in a suburban area. Both schools, especially the city school, performed academically above the national average. Active parental consent was required for participation. Ethical approval was provided by the Regional Ethics Committee of Stockholm (Ref. No. 2009/857-31/4). The present study used data collected through questionnaires and saliva.

The classroom questionnaire was completed by 413 students (76%). The internal attrition was low (0–4% for single items). Two cases with incomplete data were removed from the analyses, resulting in a study sample of 411 students (boys: *n* = 167, girls: *n* = 244). Students who completed the questionnaire were then invited to participate in a cortisol study where they sampled saliva at four time points during an ordinary school day: (a) Immediately at waking up, (b) at 30 min post-awakening, (c) at 60 min post-awakening, and (d) at 8:00 p.m. They also completed a diary during the sampling day, with questions regarding the time and date for saliva sampling, chronic diseases and medication, menarche and health behaviours. About half of the students participated in the saliva sampling. Three participants who took their first sample more than 15 min after waking up were excluded [29]. The cortisol subsample included 198 students (boys: *n* = 64; girls: *n* = 134) with information on the three morning saliva samples and 191 students with information on all four saliva samples. 

Saliva samples were collected using the Salivette^®^ sampling device (Sarstedt Inc., Rommelsdorf, Germany), a plastic tube with a suspended insert containing a sterile cotton swab. Participants were instructed to chew on the swab for two min before putting it back into the tube and sealing it, or to actively spit saliva directly into the tube, if preferred. They were instructed not to eat, smoke, drink coffee/tea or brush their teeth 30 min before providing a saliva sample. The samples were stored at room temperature until the next school day when they were returned to the research team, and stored in freezer (−20 °C) until laboratory analysed for cortisol using competitive radioimmunoassay (Spectria Cortisol RIA, Orion Diagnostica, Espoo, Finland; intra-assay precision <5%, 1.7–4.1% and interassay precision <10%, 4.3–9.0%). Each sample was analysed twice and in randomised order. For more detailed information on the study design and procedure, see elsewhere [18,30,31].

### 2.2. Measures 

Demands were captured by three items addressing the students’ perceptions of their school workload and study pace: ‘I have a lot of schoolwork’, ‘I think schoolwork is difficult’, and ‘I think it is a fast pace in school’. The response alternatives were on a five-point scale, ranging from *never* (1) to *always* (5).

Coping resources were assessed with the following three statements: ‘I receive information in good time about things that concern me as a student’, ‘I know what I need to do to get good grades’, and ‘I get the help I need from teachers in school’. All items were rated on a five-point scale ranging from *never* (1) to *always* (5).

The factor structure of the school demands and coping resources items were examined using Mplus Version 6 [32], with all items treated as ordered-categorical measures. Results showed an excellent fit for the two-factor model (*x*^2^ = 19.67, *df* = 8, *p* < 0.05, CFI = 0.99, TLI = 0.98, RMSEA = 0.06) and good internal reliability (Cronbach’s alpha: Demands = 0.74 and coping resources = 0.70). Measures of demands and coping resources respectively (*r* = −0.18) were formed by calculating the mean score of these scales if participants had responded to at least two out of three items within a scale. 

Perceived stress was measured using the Pressure-Activation Stress (PAS) Scale [33]. The instrument comprises of 11 statements describing two components of stress: *Activation* (e.g., ‘I rush even if I don’t have to’), and *pressure* (e.g., ‘I never feel really free’). Response alternatives ranged from *never* to *always* on a five-point scale. Previous research using this instrument has reported good factor structure and internal reliability of the activation and pressure subscales [33,34]. The current study also observed excellent model fit for the two-factor structure (*x*^2^ = 199.36, *df* = 43, *p* < 0.05, CFI = 0.96, TLI = 0.95, RMSEA = 0.09) and good internal reliability (Cronbach’s alpha: Activation = 0.77 and pressure = 0.80). Measures of activation and pressure were formed by calculating the mean score of these scales if participants had responded to at least 60% of items within each subscale. 

Recurrent pain was based on the question ‘How often during the last six months have you had the following problems?’: ‘Headache’, ‘Stomach-ache’, ‘Backache’, and ‘Pain in neck and shoulders’. The five response alternatives were *every day*, *several times a week*, *once a week*, *sometime during the month*, and *more* *seldom or never*. The co-occurrence of pain has been suggested to indicate that stress is a likely cause [35], and students who reported having at least two types of pain every day or several times a week were classified as experiencing recurrent pain [23].

Cortisol was analysed using two aggregate measures: ‘Area under the curve with respect to ground’ (AUC_G_), measuring the total cortisol output during a day (using samples 1–4); and ‘Cortisol awakening response with respect to ground’ (CAR_G_), measuring the cortisol output during the first hour after waking up (using samples 1–3). Raw, untransformed values of cortisol concentration (nmol/L) and the trapezoid formula developed by Pruessner et al. [36] was used to calculate both AUC_G_ and CAR_G_. The formula for AUC_G_ is:

(1) $${\mathrm{AUC}}_{G}=\sum_{i=1}^{n-1}\frac{\left(m_{\left(i+1\right)}+m_{i}\right)\cdot t_{i}}{2},$$

where *t_i_* indicates the time between cortisol measurements, *m_i_* the individual measurement, and *n* the number of measurements. Due to skewness, aggregate measures were log-transformed prior to regression analyses.

Covariates included, in addition to gender, grade (8 or 9) and school (suburban or city). For the cortisol subsample, the time waking up and the time between waking and the first saliva sample were also included as covariates.

### 2.3. Statistical Analysis

Linear (OLS) regression, presenting unstandardised b coefficients, was used in the analyses of perceived stress (pressure and activation) and cortisol (lnAUC_G_ and lnCAR_G_). Binary logistic regression, presenting odds ratios (OR), was used in the analysis of recurrent pain. All analyses were performed in SPSS (vers. 25, SPSS Inc., Chicago, IL, USA). For each outcome, Model 1 presents crude estimates of demands and coping resources respectively, adjusting for gender, grade and school. Model 2 included demands and coping resources simultaneously, in addition to control variables. The cortisol analyses also control for time of waking and the time difference between waking and the first saliva sample. Interactions between demands and coping resources were examined, as well as interactions between demands and coping resources with gender. The small variation in sample size between self-reported outcomes (reported in Table 1) reflects differences in internal non-response.

## 3. Results

Table 1 provides descriptive statistics and shows that girls reported higher demands than boys, whereas no gender difference was observed for coping resources. Girls had greater stress than boys according to all five stress-related measures: Pressure, activation, recurrent pain, and cortisol output in terms of lnAUC_G_ and lnCAR_G._ The gender difference in lnAUC_G_ was however not statistically significant at the 5%-level. 

Next, the associations between demands and coping resources and the different measures of stress were examined, with results in Table 2. In the crude models (including covariates), higher demands were associated with higher pressure (b = 0.52, *p* < 0.001), and activation scores (b = 0.42, *p* < 0.001), as well as with an increased risk of recurrent pain (OR = 1.56, *p* < 0.05), and lower levels of morning cortisol (lnCAR_G_) (b = −0.09, *p* < 0.05). Greater coping resources were associated with both lower pressure (b = −0.44, *p* < 0.001) and activation scores (b = −0.29, *p* < 0.001), and with a decreased risk of recurrent pain (OR = 0.53, *p* < 0.01). However, there were no statistically significant associations between coping resources and cortisol levels. 

In the fully adjusted models, these patterns hold suggesting independent effects of demands and coping resources. However, two estimates were no longer statistically significant at the 5%-level, i.e., the associations between demands and recurrent pain (OR = 1.44, *p* < 0.10), and morning cortisol (lnCAR_G_; b = −0.09, *p* < 0.10) respectively. 

The gender difference with greater stress among girls is visible for all stress-related outcomes also in Table 2. For each outcome, we tested for a set of interactions to investigate potential gender differences in the associations between demands and coping resources and each dependent variable. Statistically significant interactions between gender and demands were observed for activation and for recurrent pain. The gender-separate analyses (see lower part of Table 2) demonstrate that demands were more strongly and positively associated with activation among girls (b = 0.53, *p* < 0.001) than among boys (b = 0.25, *p* < 0.01), and with recurrent pain only among girls (OR = 2.38, *p* < 0.01), but not among boys (OR = 0.57, ns). 

The interactions between gender and coping resources were statistically significant for pressure and for activation. The gender-stratified analyses (see lower part of Table 2) show that coping resources were more strongly associated with pressure and activation for boys, than for girls (pressure: Boys b = −0.50, *p* < 0.001, girls b = −0.19, *p* < 0.01; activation: Boys: b = −0.38, *p* < 0.001, girls b = −0.05, ns). 

For each outcome, we also tested for interactions to assess the combination of demands and coping resources. An interaction between demands and coping resources was statistically significant only for recurrent pain, and in the gender-separate analyses only among boys. Further analyses (data not presented) showed that boys with both lower levels of demands and of coping resources had the highest likelihood of reporting recurrent pain. 

## 4. Discussion

Previous research has shown that many adolescents, particularly girls, report high levels of stress-related health complaints [5,6], with school demands having been identified as one central stressor in the lives of adolescents [7,8,9,10,11,12,13,14,15,16]. Thus, examining if there are school-based resources that may help students to reduce their experiences of stress is a relevant task. 

The aim of this study was to examine the associations between task-related demands and task-related coping resources at school with different measures of stress among students, and to scrutinise potential gender differences. Using data from a multiple methods data collection, we investigated stress in terms of self-report measures of perceived stress and recurrent pain, and biomarkers in terms of salivary cortisol. Reflecting previous research [5,6], girls reported higher levels of perceived stress and recurrent pain than did boys. Girls also had higher cortisol output compared to boys, which may partly reflect biological differences and variation in pubertal maturation between genders [18,30]. Furthermore, girls experienced higher levels of demands compared to boys, whereas there was no gender difference in access to coping resources.

In line with earlier findings [7,8,9,10,11,12,13,14,15,16], higher demands were associated with greater perceived stress and an increased risk of recurrent pain. The result that higher levels of demands were associated with a lower morning cortisol response may seem counterintuitive, but align with previous studies reporting that chronic stressors, including exposure to bullying, are linked to lower cortisol levels [23]. Thus, it is possible that prolonged experiences of excessive school-related demands yield chronic stress, which over time impacts the functioning of different physiological systems, such as the cardiovascular, immune, and metabolic systems [4], but also affects the HPA-axis. This is perhaps what is reflected in the lower levels of morning cortisol. In contrast to demands, greater coping resources at school were clearly associated with less perceived stress and a lower likelihood of recurrent pain. Yet, there was no association between coping resources and cortisol output, suggesting no protective effects of school-based control and support on stress as reflected in the cortisol measures. This study also investigated combinations of demands and coping resources, and was particularly interested in whether or not access to coping resources may buffer the effect of high demands on stress. However, no support for such an interaction was found. The only statistically significant interaction effect indicated that low demands and low coping resources were connected with a particularly high likelihood of reporting recurrent pain among boys.

Several gender differences were found in relation to demands and coping resources. Apart from greater demands being more common among girls, they were also more strongly linked to greater perceived stress in terms of activation among girls than among boys, and were associated with an increased likelihood of recurrent pain among girls, but not among boys. In contrast, the access to coping resources were of similar magnitude among boys and girls, and the protective effect of coping resources on perceived stress was more pronounced among boys than girls. 

The findings that girls reported higher levels of demands than boys, and that the association between demands and stress was especially pronounced among girls, deserve attention. Giota and Gustafsson [16] make note of the seemingly paradoxical finding that girls perceive higher school demands than boys, despite their on average higher achievement. They suggest that one explanation may be that girls are less inclined to feel that they live up to the demands posed on them, which also leads to more stress. They also discuss the possibility that girls might need higher educational success in order to compete on the labour market [16]. Another possible interpretation is that gendered norms regarding academic achievement result in girls on average having higher academic aspirations than boys, and that girls and boys therefore have different views of and attitudes towards school demands and what is ‘good enough’. This interpretation is supported by a qualitative study based on the same project as the present study. According to interviews conducted with grade 8 students, boys seemed to put less effort into schoolwork, were more relaxed towards schoolwork and their performance, and also were more satisfied with lower marks than the girls [30]. Thus, girls have higher levels of demands and stress than boys, and the stress levels of girls also appear less easily reduced through coping resources. So, in line with Giota and Gustafsson [16], it seems important to identify the specific features of the schoolwork that give rise to stress and to act in relation to these features to successfully decrease stress among students.

The fact that stress among boys was clearly associated with coping resources, and more so than among girls, also deserves attention. Greater coping resources were linked with less stress in terms of both perceived stress and recurrent pain. It is possible that control may be less important in the face of very high demands, but more important at lower levels of demands. This might partly explain why coping resources generally are more important for boys. Furthermore, access to coping resources, in this study, means having timely information of importance to students, knowledge about how to get good grades, and getting help from teachers when required. These resources are important not only in order to perform well in terms of academic achievement, but also to fulfil one’s duties as a student more generally, irrespective of one’s academic ambitions. In other words, not aiming for the highest possible marks does not mean that the student neglects schoolwork per se.

With regard to coping resources, earlier studies have shown that teacher support is linked to students’ health [7,8]. In the present study, the association between the coping resources index and stress outcomes was, however, not mainly driven by teacher support. Additional analyses showed that students’ access to information in good time was the type of coping resource most clearly and negatively associated with pressure, activation, and recurrent pain (data not shown). To provide timely information of relevance to students may thus be one important way for schools to promote student health.

As regards the present study, its main strength lies in the fact that it included both self-report measures of perceived stress and recurrent pain, and physiological measures of stress. Indeed, to measure stress among adolescents, using multiple methods has been recommended [24]. Yet, there are limitations, which for instance include the relatively small sample, especially the cortisol subsample, and in particular when stratifying by gender, which potentially reduces statistical power. Moreover, the sample included students performing above the national average. This was true for both schools, but particularly the city school had high-performing students (and a higher reporting of pressure and recurrent pain according to Table 2). In addition, both schools had an overrepresentation of students with parents with a tertiary education, as compared to the national average [30]. With highly educated parents, it seems likely that many of the students in our sample received support from their parents in their schoolwork. It is possible that such support decreased students’ experiences of school demands, and/or attenuated the strength of the association between demands or coping resources and stress. Thus, to make generalisations, further studies, including students with other sociodemographic characteristics are needed. Finally, the data were cross-sectional, which allows no empirical examination of causality, meaning that longitudinal studies of demands, coping resources and multiple measures of stress are recommended. Data with repeated measures from several points in time would also make it possible to assess the potential cumulative effects of demands and coping resources on self-reported stress and physiological stress markers, and ideally reflect the functioning of different systems [4], to also include the immune system.

## 5. Conclusions

School demands have been shown to constitute an important stressor among adolescents. The present study confirmed this and also demonstrated that greater school-based coping resources were related to less stress among students, especially boys. Thus, the findings clearly showed that schools, through their timely dissemination of relevant information to students and through teacher support, can promote students’ coping resources. Nevertheless, while schools can reduce student stress by promoting coping resources, identifying specific features of the schoolwork that give rise to stress and to modify these accordingly is obviously important as well.

## Figures and Tables

**Table 1:** Descriptive statistics of the sample and study variables.

	Study Sample						Cortisol Subsample		
		Demands	Coping Resources	Pressure	Activation	Recurrent Pain		lnAUC_G_	lnCAR_G_
	%	Mean	Mean	Mean	Mean	%	%	Mean	Mean
*Gender*									
Boys	41	3.37	3.83	2.69	2.53	10.2	32	8.87	6.78
Girls	59	3.61 **	3.77	3.33 ***	3.11 ***	22.7 **	68	9.02 ^†^	7.07 ***
*Grade*									
8	51	3.47	3.80	3.04	2.86	17.2	57	8.95	6.98
9	49	3.55	3.79	3.12	2.89	17.9	43	9.00	6.96
*School*									
Suburban	30	3.50	3.83	2.89	2.72	10.7	25	8.96	6.95
City	70	3.52	3.78	3.16 **	2.94 *	20.5 *	75	8.97	6.98
*All*	100	3.51	3.79	3.08	2.87	17.6	100	8.97	6.97
*n*	411	411	411	407	406	404	198	191	198

*** *p* < 0.001, ** *p* < 0.01, * *p* < 0.05, ^†^ *p* < 0.10. *Note. T*-tests performed for all variables except recurrent pain; chi-square performed for recurrent pain.

**Table 2:** Results from linear (OLS) regressions of pressure, activation and cortisol output, and from binary logistic regressions of recurrent pain, by demands and coping resources in school.

	Study Sample *n* = 404–407						Cortisol Subsample *n* = 191–198			
	Pressure		Activation		Recurrent Pain		lnAUC_G_		lnCAR_G_	
	b	b	b	b	OR	OR	b	b	b	b
	Model 1	Model 2	Model 1	Model 2	Model 1	Model 2	Model 1	Model 2	Model 1	Model 2
*Whole sample*										
Demands	0.52 ***	0.47 ***	0.42 ***	0.39 ***	1.56 *	1.44 ^†^	−0.06	−0.06	−0.09 *	−0.09 ^†^
Coping resources	−0.44 ***	−0.35 ***	−0.29 ***	−0.22 ***	0.53 **	0.57 **	0.00	−0.01	0.03	0.01
Gender (girls)	0.62 ***	0.48 ***	0.56 ***	0.46 ***	2.40 **	2.28 **	0.15 ^†^	0.16 *	0.29 ***	0.31 ***
Grade (9)	0.08	0.04	0.03	0.00	1.07	1.05	0.07	0.06	−0.03	−0.03
School (city)	0.20 *	0.19 **	0.15	0.15 ^†^	1.94 *	1.94 ^†^	0.03	0.02	0.02	0.02
*Interaction Gender-Demands*		*ns*		*s*		*s*		*ns*		*ns*
*Interaction Gender-Coping resources*		*s*		*s*		*ns*		*ns*		*ns*
*Interaction Demands-Coping resources*		*ns*		*ns*		*s*		*ns*		*ns*
*Girls*										
Demands	0.56 ***	0.53 ***	0.53 ***	0.53 ***	2.43 **	2.38 **	−0.06	−0.07	−0.07	−0.07
Coping resources	−0.28 ***	−0.19 **	−0.14	−0.05	0.65	0.71	−0.06	−0.07	−0.01	−0.02
*Interaction Demands-Coping resources*		*ns*		*ns*		*ns*		*ns*		*ns*
*Boys*										
Demands	0.48 ***	0.38 ***	0.31 **	0.25 **	0.69	0.57	−0.03	−0.02	−0.12 ^†^	−0.11
Coping resources	−0.57 ***	−0.50 ***	−0.42 ***	−0.38 ***	0.43 **	0.38 **	0.02	0.01	0.06	0.02
*Interaction Demands-Coping resources*		*ns*		*ns*		*s*		*ns*		*ns*

*** *p* < 0.001, ** *p* < 0.01, * *p* < 0.05, ^†^ *p* < 0.10. *Note.* Model(s) 1 controls for gender, grade and school, and when analysing cortisol also for awakening time and time difference between awakening and first sample; Model(s) 2 includes both demands and coping resources in addition to the control variables (interaction terms not included).
